# Association between body mass index and health-related quality of life among Chinese elderly—evidence from a community-based study

**DOI:** 10.1186/s12889-018-6086-1

**Published:** 2018-10-12

**Authors:** Hua You, Xiao-lu Li, Kang-zhen Jing, Zhi-guang Li, Hong-mei Cao, Jin Wang, Lan Bai, Jing-hong Gu, Xiaoman Fan, Hai Gu

**Affiliations:** 1 Department of Social Medicine and Health Education, School of Public Health, Nanjing Medical University, 101 Longmian Ave, Nanjing, 211166 China; 2 Center for Health Policy and Management Studies, Nanjing University, 22 Hankou Road, Nanjing, 210093 China; 3 The First Affiliated Hospital with Nanjing Medical University, 300 Guangzhou Road, Nanjing, 210029 China; 4 The Affiliated Drum Tower Hospital of Nanjing University Medical School, 321 Zhongshan Road, Nanjing, 210008 China; 5 Nanjing Foreign Language School, Nanjing, 210018 China

**Keywords:** Body mass index, Quality of life, Elderly

## Abstract

**Background:**

This study aimed to explore the effects of (body mass index) BMI on health related quality of life (HRQoL) among the elderly in Jiangsu, China.

**Methods:**

A total of 10,257 community dwelling elderly (≥60 years old) were enrolled in a cross-sectional study. HRQoL was measured via the Eq-5d-3 L. Chi-square tests and one-way ANOVA analyses were used to compare the frequencies and scores of Eq-5d responses among different BMI groups (defined as “underweight”, “normal weight”, “overweight” and “obese”). Logistic regression analyses were conducted to examine the associations between BMI and HRQoL.

**Results:**

Among the subjects, the proportion of “normal weight”, “underweight”, “overweight” and “obese” were 66.0, 8.3, 23.1, and 2.6%, respectively. The score of the Eq-5d index among total participants was 0.8036 and the Visual Analog Scale (VAS) score was 75.47. For both the responses frequency and scores of Eq-5d-3 L, there were significant differences among BMI groups (*P* < 0.001). The Logistic regression model showed that both in men and women, underweight elderly were more likely to suffer low HRQoL. The adjusted odds ratio (OR) with a 95% confidence interval (CI) for Eq-5d index/VAS was 2.03 (1.48, 2.79)/1.83 (1.34, 2.50) in men and 1.47(1.09,1.98)/1.52(1.20,1.91) in women. Overweight women more likely to have a low Eq-5d index, while overweight men were less likely to have a low Eq-5d VAS.

**Conclusion:**

This study shows that underweight is an explicit risk factor of low HRQoL in both the male and female elderly, while the effect of overweight on low HRQoL varies slightly by gender.

## Background

A rising global epidemic of overweight and obesity is taking over many parts of the world. As of 2016, the number of overweight adults had increased to more than 1.9 billion, and of these over 600 million were obese [1]. Overweight and obese persons are at risk of a number of medical conditions which can lead to further morbidity and mortality [2]. In China, the number of overweight and obese people is close to a quarter of the total population [3]. A recent study based on China Health and Nutrition Survey reported the rate of adult obesity to be 12% among men and 11% among women [4]. Hence, the problem of overweight and obesity is a critical public health issue in the country.

From the obesity perspective, there is a growing interest in the health-related quality of life(HRQoL). HRQoL is a difficult concept and comprises of multi-dimensional constructs that includes self-assessment of physical, emotional and social well-being [5]. There is growing evidence that obesity has a detrimental effect on HRQoL [6] . Even after controlling for important social-demographic features and health-risk behaviors, deviations from normal weight are associated with compromised physical or mental HRQoL [7]. However, some early studies also reported that there was no significant association between a high BMI and a low quality of life, especially in Asian population [8, 9]. In addition to the relationship between obesity and HRQoL, the potential effect of underweight on HRQoL is often neglected, even though the underweight individuals may also suffer from compromised HRQoL [10].

As China rapidly transitions into an ageing society with over 200 million people aged over 60 years [11], the disease burden attributable to unhealthy body weight is expected to increase. The growing rates of abnormal body weight may have an important influence on the quality of life in this large number of elderly people, as unhealthy body weight aggravates disease-related symptoms [9]. However, Chinese research in this field is lacking. So far, though there are some studies examining the association between body weight and HRQoL, most of them have been conducted in western or other high income countries. Furthermore, those studies often relied upon clinical samples, which might have led to an overestimation of the adverse relationship between excessive weight on HRQoL [7]. The influence of body weight on HRQoL among the community-residing elderly in developing countries has been rarely studied.

The main aim of the present study was to examine the association between HRQoL and BMI (ranging from underweight to obesity) in a community-dwelling elderly sample aged over 60 years in Jiangsu, China. In particular, this study examined the relationship between BMI category and the level of HRQoL in elderly males and females.

## Methods

### Study design and data collection

Data for this study was derived from the fifth National Health Services Survey 2013 conducted in Jiangsu province(J-NHSS) that is located in eastern coastal area of China, and has a population of 79.38 million (2013) with top level the socio-economic indicators among all provinces in China.

The 2013 J-NHSS adopted a multistage stratified random sampling strategy. First, the counties (rural areas)/ districts (urban areas) were chosen randomly. Second, towns were drawn from each county/district, and then villages/communities were drawn randomly from each town. Finally, considering diversities in geographic location and economic status, the number of households drawn from each village/community was determined according to the proportion of the number of households (details of the NHSS design have been published [12]).The J-NHSS covered 18 counties (rural areas) or districts (urban areas) of Jiangsu. A total of 12,600 households from 180 villages/communities that were from 90 towns were selected to participate in the survey.

A questionnaire was administered to the selected participants by interviewers recruited from local health workers. The investigations were conducted by professionals after written consent forms (consent to participate under the ‘Ethics, Consent and Permissions’ heading and to publish their data in this study in any form) were explained and accepted. Ethical approval was obtained from the Ethics Committee of Medical Faculty, Nanjing Medical University.

A total of 36,381 individuals (from 12,600 households) in Jiangsu province completed the survey in 2013. For the purpose of this study, a final sample of 10,257 individuals aged ≥60 years were enrolled. Exclusion criteria for individuals were cognitive or awareness problems, and the inability to understand or answer the questions.

### Measurement of HRQoL

Eq-5d-3 L is preselected for the measurement of an individual’s self-perceived HRQoL in the NHSS. It is a five-item questionnaire encompassing the domains of mobility, self-care, usual activities, pain/discomfort, and anxiety/depression, as well as overall health rated on a Visual Analog Scale (VAS) [13, 14]. For the above five dimensions, each has 3 response levels (i.e., 1 = no problems, 2 = some/moderate problems, and 3 = extreme problems). This allows for 243 possible combinations of health conditions. HRQoL results measured by the Eq-5d-3 L were converted to an index score (a proxy health utility score) using the Japan Time-Trade Off (TTO) value set which ranges from − 0.11 to 1.00 [15], in which a score close to 1 indicates better the health. In contrast, for each domain, a higher score indicates a more serious problem. The Eq-VAS scores ranged from 0 (worst health) to 100 (best health). The equivalence between the Chinese version and the English version of EQ-5d-3 L has been proved [16]. Also, the Chinese version has passed the test of reliability and validity both in previous studies [17] and this study. The Cronbach’s α of EQ-5d index in this analysis was 0.852. The Correlation coefficient between VAS and index score was 0.540 (*P* < 0.05); the Correlation coefficients for VAS and the five domains were − 0.472,-0.422,-0.470,-0.477 and − 0.378 (*P* < 0.05), respectively.

### Body mass index

All respondents were asked to report their height and weight. BMI was calculated as the weight (in kilograms) divided by the square of the height (in meters) (kg/m2). The study subjects were classified into “underweight” (< 18.5), “normal weight” (18.5–24.0), “overweight” (24.0–28.0) and “obese” (≥28.0), based on the BMI quartiles which were formed according to the “Criteria of Weight for Chinese Adults” [18].

### Control variables

Three categories of control variables were included in the analyses. The first category was demographic characteristics, including age, marriage, education, registered residence, and income. The second category was health related behaviors, including smoking (at present or in the past), alcohol consumption in the last 12 months, and physical exercise (in the last 6 months, when the average weekly exercise exceeded once per week). The third category inquired about two kinds of health conditions: whether there was a diagnosis with any chronic disease or not and whether there was a hospitalization or not in the last 12 months.

### Statistical analyses

All statistical analyses were performed using SPSS for windows (version 18.0, SPSS, Inc., Chicago, IL). Chi-square tests were used to compare the frequencies of Eq-5d responses among the different BMI groups. Then, One-way ANOVA analyses were selected to compare the scores among BMI groups. Additionally, Post Hoc Multiple Comparisons were also conducted using the Dunnett’s T3 methods, since equal variances were not assumed.

Binary Logistic regressions were used to examine the influence of BMI on Eq-5d scores. The dependent variables were divided into two categories depending on whether the Eq-5d score (Eq-5d index in model I-1 and I-2, Eq-5d VAS in model II-1 and II-2) was lower than the Chinese elderly norm. A previous study reported the norm value of EQ-5d-3 L index/VAS among Chinese population ≥ 60 years. The norm value of index/VAS for males was 0.82/76.95, and for females was 0.73/73.55 [19]. According to the research objective, BMI groups were the core independent variable in these regression models. In addition, the control variables were adapted including demographic characteristics, health related behaviors and health conditions.

## Results

### Characteristics of the study population

The Characteristics of participating older adults are shown in Table 1. The average age was 69.57 years (SD = 7.5), and 5058(49.3%) were males. Approximately 21.0% were not married. For health-related behaviors, 14.5% of participants currently smoked, 23.7% drank alcohol in the past year, and 32.2% did physical exercise every week for the past 6 months. The average BMI was 22.87 kg/m^2^ (SD = 3.4). The proportion of normal weight, underweight, overweight and obese was 66.0, 8.3, 23.1, and 2.6%, respectively.

**Table 1:** General characteristics according to gender

Characteristics	Male(5058)	Female(5199)	Total(10257)
	Mean(SD)/n%	Mean(SD)/n%	Mean(SD)/n%
Age(10257)	69.25(7.0)	69.89(7.9)	69.57(7.5)
Marital status(10257)			
Married	4396(86.9)	3079(71.3)	8105(79.0)
Not married(unmarried, divorced and widowed)	662(13.1)	1490(28.7)	2152(21.0)
Educational level(10257)			
Below high school	4223(83.5)	4840(93.1)	9063(88.4)
High school and above	835(16.5)	359(6.9)	1194(11.6)
Registered residence(10257)			
Rural	2348(46.4)	2655(51.1)	5003(48.8)
Urban	2710(53.6)	2544(48.9)	5254(51.2)
Household income per capita(10162)	$2464.99(2899.40)	$2421.88(2362.40)	$2443.15(2641.05)
Income group(10162)			
≤ $935.54	1004(20.0)	1096(21.3)	2100(20.7)
$935.54~$1461.78	1040(20.7)	1037(20.1)	2077(20.4)
$1461.78~$23,388.48	1081(21.6)	1064(20.7)	2145(21.1)
$23,388.48~$3508.27	900(17.9)	937(18.2)	1837(18.1)
>$3508.27	990(19.7)	1013(19.7)	2003(19.7)
Smoking(7835)			
Yes	1047(35.6)	87(1.8)	1134(14.5)
No	1891(64.4)	4810(98.2)	6701(85.5)
Alcohol consumption(10186)			
Yes	2932(58.2)	311(6.0)	2419(23.7)
No	2108(41.8)	4835(94.0)	7767(76.3)
Physical exercise(10240)			
Yes	1715(33.9)	1586(30.6)	3301(32.2)
No	3338(66.1)	3601(69.4)	6939(67.8)
Chronic disease(10257)			
With	2633(52.1)	2839(54.6)	5472(53.3)
Without	2425(47.9)	2360(45.4)	4788(46.7)
Hospitalization(10257)			
Yes	639(13.0)	538(10.7)	1177(11.5)
No	4268(87.0)	4475(89.3)	8743(85.2)
BMI (10245)	22.76(3.2)	22.97(3.5)	22.87(3.4)
BMI group(10245)			
Normal weight	2981(59.0)	2833(54.5)	6761(66.0)
Underweight	400(7.9)	455(8.8)	855(8.3)
Overweight	1416(28.0)	1521(29.3)	2367(23.1)
Obese	254(5.0)	385(7.4)	262(2.6)

Income has been converted to dollars at exchange rates and been grouped according to quintile

### HRQoL among different BMI groups

As shown in Table 2 and Table 3, both for responses frequency and scores of Eq-5d, there were significant differences among different body weight groups (*P* < 0.001). Furthermore, significant differences were observed in each dimension where the numbers of elderly with major problems were bigger in underweight groups than in the other two groups (Table 2). From the Eq-5d scores (Table 3), the results of one-way ANOVA disclosed the significant differences among four BMI groups in all five dimensions (*P* < 0.001), as well as in the Eq-5d index (*P* < 0.001) and VAS (*P* < 0.001). For each dimension, the score of the underweight group was the highest (a higher score in each dimension means lower health as the negative measurement), and the results of the Post Hoc Multiple Comparisons showed it to be significantly different from the score of the normal weight group (*P* < 0.001). Both for the Eq-5d index and VAS, scores of the underweight group were the lowest, and were significantly lower than the scores of the normal group (0.7616 VS. 0.8067, *P* < 0.001; 69.92 VS. 76.89, *P* < 0.001). However, there were no significant differences between scores of the normal group and overweight/obese group, with respect to the Eq-5d index, VAS or each dimension score (*P* > 0.05). The score of Eq-5d index among total participants was 0.8036 and the VAS was 75.47 (Table 3).

**Table 2:** The frequency distribution of response to Eq-5d according to BMI groups, n(%)

Dimensions	BMI groups				Total
	Underweight	Normal Weight	Overweight	Obese	
*Mobility*					
No problems	680(79.5)	5209(89.6)	2637(89.8)	560(87.6)	9086(88.7)
Some problems	145(17.0)	528(9.1)	281(9.6)	71(11.1)	1025(10.0)
Major problems	30(3.5)	77(1.3)	19(0.6)	8(1.3)	134(1.3)
*P*	< 0.001				
*Self-care*					
No problems	732(85.6)	5464(94.0)	2787(94.9)	603(94.4)	9586 (93.6)
Some problems	91(10.6)	252(4.3)	118(4.0)	28(4.4)	489(4.8)
Major problems	32(3.7)	98(1.7)	32(1.1)	8(1.3)	170(1.6)
*P*	< 0.001				
*Usual activities*					
No problems	676(79.1)	5285(90.9)	2693(91.7)	575(90.0)	9229(90.1)
Some problems	117(13.7)	379(6.5)	186(6.3)	49(7.7)	731(7.1)
Major problems	62(7.3)	150(2.6)	58(2.0)	15(2.3)	285(2.8)
*P*	< 0.001				
*Pain/Discomfort*					
No problems	619(72.4)	4826(83.0)	2406(81.9)	508(79.5)	8359(81.6)
Some problems	216(25.3)	942(16.2)	506(17.2)	123(19.2)	1787(17.4)
Major problems	20(2.3)	46(0.8)	25(0.9)	8(1.3)	99(1.0)
*P*	< 0.001				
*Anxiety/Depression*					
No problems	739(86.4)	5454(93.8)	2748(93.6)	600(93.9)	9541(93.1)
Some problems	108(12.6)	334(5.7)	182(6.2)	39(6.1)	663(6.5)
Major problems	8(0.9)	26(0.4)	7(0.2)	0(0.0)	41(0.4)
*P*	< 0.001				

10,245 (99.9%) were included in the above analyses as 12 were missing

**Table 3:** Comparison of Eq-5d scores among different body weight groups, Mean(SD)

Eq-5d score	BMI groups				Total
	Underweight	Normal Weight	Overweight	Obese	
*Mobility*	0.0274(0.0796)	0.0124(0.0517)	0.0099(0.0396)	0.0136(0.0513)	0.0130(0.0517)
F(*P*)					26.09(< 0.001)
*P*_1_	< 0.001				
*P*_2_			0.078		
*P*_3_				0.994	
*Self-care*	0.0096(0.0247)	0.0041(0.0169)	0.0033(0.0148)	0.0036(0.0157)	0.0043(0.0171)
F(*P*)					31.33 (< 0.001)
*P*_1_	< 0.001				
*P*_2_			0.151		
*P*_3_				0.989	
*Usual activities*	0.0157(0.0361)	0.0063(0.0233)	0.0054(0.0210)	0.0065(0.0229)	0.0068(0.0242)
F(*P*)					43.00(< 0.001)
*P*_1_	< 0.001				
*P*_2_			0.365		
*P*_3_				1.00	
*Pain/Discomfort*	0.0248(0.0434)	0.0145(0.0335)	0.0154(0.0344)	0.0178(0.0372)	0.0158(0.0350)
F(*P*)					22.19(< 0.001)
*P*_1_	< 0.001				
*P*_2_			0.783		
*P*_3_				0.171	
*Anxiety/Depression*	0.0090(0.0232)	0.0041(0.0163)	0.0041(0.0161)	0.0039(0.0151)	0.0045(0.0169)
F(*P*)					21.98(< 0.001)
*P*_1_	< 0.001				
*P*_2_			1.00		
*P*_3_				1.00	
*Eq-5d index*	0.7616(0.1708)	0.8067(0.1146)	0.8098(0.0990)	0.8026(0.1115)	0.8036(0.1168)
F(*P*)					41.43(< 0.001)
*P*_1_	< 0.001				
*P*_2_			0.705		
*P*_3_				0.945	
*Eq-5d VAS*	69.92(14.73)	76.89(12.63)	76.34(12.86)	75.08(13.30)	75.47(13.03)
F(*P*)					59.20(< 0.001)
*P*_1_	< 0.001				
*P*_2_			0.526		
*P*_3_				0.605	

*P*_*1*_ :*P* for underweight group VS. normal weight group, *P*_2_ :*P* for overweight group VS. normal weight group, *P*_3_ :*P* for obese group VS. normal weight group

### The influence of abnormal weight on Eq-5d scores

The results of Logistic regression showed the association between abnormal body weight and Eq-5d score (Table 4)/VAS (Table 5) below the Chinese elderly norm value. For elderly males, the underweight group was more likely to have a low Eq-5d index (adjusted OR = 2.03, 95% CI = 1.48, 2.79) and VAS (adjusted OR = 1.83, 95% CI = 1.34, 2.50); for elderly females, the results of the underweight group were similar for index (adjusted OR = 1.47, 95% CI = 1.09, 1.98) and VAS (adjusted OR = 1.52, 95% CI = 1.20, 1.91). Overweight elderly females were more likely to have a low index (adjusted OR = 1.33, 95%CI = 1.08, 1.65), while the overweight male group was less likely to have low VAS (adjusted OR = 0.78, 95% CI = 0.65, 0.95).

**Table 4:** The influence of abnormal weight on Eq-5d index below the Chinese elderly norm

BMI group	Model I-1 (Male)		Model I-2 (Female)	
	Crude OR(95%CI)	Adjusted OR ^a^ (95%CI)	Crude OR(95%CI)	Adjusted OR ^a^ (95%CI)
Normal Weight	1	1	1	1
Underweight	2.26(1.81,2.83)***	2.03(1.48,2.79)***	2.18(1.72,2.78)***	1.47(1.09,1.98)*
Overweight	0.90(0.77,1.06)	0.99(0.80,1.24)	1.10(0.91,1.31)	1.33(1.08,1.65) **
Obese	1.01(0.73,1.39)	1.01(0.65,1.59)	1.22(0.91,1.65)	1.19(0.83,1.68)

^a^ Adjusted for demographic characteristics (age, marriage, education, registered residence, income), health related behaviors (smoking, alcohol consumption, physical exercise), and health conditions (chronic diseases and hospitalization)

2794 (55.2% of male cases) were included in Model I-1 and 4615(88.8% of female cases) were included in Model I-2

* *p* < 0.05; ** *p* < 0.01; *** *p* < 0.001

**Table 5:** The influence of abnormal weight on Eq-5d VAS below the Chinese elderly norm

BMI group	ModelII-1(Male)		ModelII-2(Female)	
	Crude OR(95%CI)	Adjusted OR^a^ (95%CI)	Crude OR(95%CI)	Adjusted OR^a^ (95%CI)
Normal Weight	1	1	1	1
Underweight	2.06(1.67,2.54)***	1.83(1.34,2.50)***	1.89(1.55,2.31)***	1.52(1.20,1.91)***
Overweight	0.88(0.77,0.99)*	0.78(0.65,0.95) *	1.02(0.90,1.16)	1.03(0.89,1.19)
Obese	0.85(0.65,1.11)	0.76(0.52,1.11)	1.25(1.01,1.55) *	1.12(0.88,1.42)

^a^ Adjusted for demographic characteristics (age, marriage, education, registered residence, income), health related behaviors (smoking, alcohol consumption, physical exercise), and health conditions (chronic diseases and hospitalization)

2794 (55.2% of male cases) were included in Model II-1 and 4615(88.8% of female cases) were included in Model II-2

* *p* < 0.05; ** *p* < 0.01; *** *p* < 0.001

The Hosmer-Lemeshow (H-L) test showed a good degree of fit in each model (*P* = 0.56, 0.61, 0.79, 0.18 for model I-1, I-2, II-1, II-2).

## Discussion

This study indicated that underweight was associated with low HRQoL among the Chinese elderly population, nevertheless the HRQoL was lowest among underweight elderly than others. Meanwhile, the results uncovered the different impacts of overweight on HRQoL for men and women: overweight positively influenced HRQoL among elderly males, while negatively impacted HRQoL among elderly females. In addition, this study did not find that obesity has a statistically significant effect on HRQoL.Our findings in relation to weight status were not in keeping with other studies conducted in old population. A previous study in Korea reported that a higher obesity level is related to a lower quality of life [20]. Another study conducted in California reported that obese older adults tend to have lower HRQOL than those who are overweight or of normal BMI [21]. Further, in a study among elderly subjects in Spain, the quality of life score was lower in obese subjects than in the normal weight group for both men and women [22].

Though the related reports almost entirely focus on obese individuals, underweight should not be ignored. There was some evidences proving that underweight was associated with increased mortality [23]. However, the relationship between underweight and quality of life has not received enough attention, even though underweight individuals also suffer from compromised HRQoL [7]. Several studies conducted in the general adult population reported underweight persons also had lower HRQoL scores like persons with excess weight [10]. Another study conducted with young men found being underweight predicted reduced HRQoL, mainly in the mental domain7 [9]. One study in China indicated that for long-lived women (over 95 years of age), low BMI, rather than elevated BMI, is an indicator of poor psychological well-being [24]. In our findings, the underweight older adults had a lower HRQoL than their normal weight counterparts, but there were nearly no differences between the obese group and the normal weight group.

There may be several potential reasons for this. Firstly, most previous studies in the field relied upon clinical samples with chronic diseases, which may have resulted in an overestimation of the strength of the association between excessive weight and HRQoL [25], while the effect of weight status on HRQoL among elderly living in the community could be different. Secondly, the number of obese subjects was relatively low (2.6% of total) in our survey sample, and the level of obesity in our subjects was not very high. Most previous studies including those conducted in the United States and Europe reported morbidly obese (BMI ≥ 35.0 kg/m^2^) subjects who had substantially lower HRQoL [9]. However, the number of morbidly obese old subjects was very small in our study, and the association between obesity and a low quality of life was not so conspicuous as the one between underweight and low HRQoL.

This study also had a noteworthy result that overweight was a risk factor for low HRQoL in women, while it was a protective factor in men. Existing literature suggests that the relationship between BMI and HRQoL is different between men and women. A study [26] found that being overweight was a risk factor of lower HRQoL in women. For men, however, being overweight was associated with higher HRQoL levels. Similarly, another study [27] using EQ-5D also found that women had a higher HRQoL burden of being overweight than men.

From the perspective of the cultural factors, most Chinese people view being heavy as a sign of health and prosperity. Moreover, it was reported that obese people were likely to have lower level of depressive symptoms, as in the so-called “Jolly Fat” hypothesis [28, 29]. A previous study in a Chinese old population had reported findings consistent with the hypothesis [30]. If excess weight is taken to be a sign of health, prosperity, and “being jolly”, then underweight may indicate poor health, poverty and “being joyless”, and thus lead to a lower quality of life. The hypothesis of “Jolly Fat” was also supported by another study [31] with older adults, which found that men’s depression was inversely proportional to their body weight. In women, the likelihood that they carry more stigma about being overweight than men may have the opposite effect. There are some policy suggestions that may be used for the implementation of public health programs. In the health management aimed at improving the quality of life for the elderly, weight management should be emphasized. Underweight elderly should be paid more attention by community health care workers, and the monitoring of low-weight elderly people should be organized within the community. These findings should be considered in the design of future weight management programs targeted towards the elderly population, including gender-specific intervention strategies.

### Limitations

The following limitations of the present study should be acknowledged. First, due to ignoring complex survey design, the results of this study may be biased and have a low *P* value. A previous study [32] using similar data proved that survey design analyses with the sampling weight should be adopt to ensure the development of appropriate estimates. However, this suggested method was not used in this research because the sample weights of the public data from NHSS were unavailable. Second, old subjects can have relatively low capabilities to accurately understand and finish the questionnaires, which may have resulted in a lower reliability of the quality of life estimates. In our study process, reviewer-administered rather than self-administered questionnaires were used to collect data which could reduce the bias introduced with the low response capabilities of the elderly [33, 34].

## Conclusions

In this cross-sectional study conducted on the elderly in communities, it appears that being underweight may be a more important contributor to compromised HRQoL than excessive weight among the Chinese elderly. It suggests that, in health care for the elder population, underweight of those whose weights are below normal should be paid more attention to by individuals and medical providers, and the overweight group should be treated distinctly between male and female older adults.

## Abbreviations

ANOVA: Analysis of variance
BMI: Body Mass Index
CDC: Centers for disease prevention and control
HRQoL: Health-related quality of life
NHSS: National health services survey
TTO: Time-trade off
VAS: Visual analog scale
